# Pharmacist Clinical Interventions and Discharge Counseling in Medical Rehabilitation Wards in a Local Hospital: A Prospective Trial

**DOI:** 10.3390/geriatrics3030053

**Published:** 2018-08-23

**Authors:** Rosanna Nga Suet Ip, Justin Wade Tenney, Angus Chun Kwok Chu, Pauline Lai Ming Chu, Grace Wai Man Young

**Affiliations:** 1 Department of Pharmacy, Tuen Mun Hospital, Hong Kong, China; chulm@ha.org.hk (P.L.M.C.); ywm201@ha.org.hk (G.W.M.Y.); 2 School of Pharmacy, The Chinese University of Hong Kong, Shatin, Hong Kong, China; tenneyjw@gmail.com; 3 Department of Medicine and Geriatrics, Tuen Mun Hospital, Hong Kong, China; chuck@ha.org.hk

**Keywords:** pharmacist, pharmacist intervention, medication reconciliation, discharge counseling, drug-related problem, readmission, unplanned health care utilization, medication adherence

## Abstract

Patients undergoing rehabilitation experience numerous changes in medication regimens during care transitions, exposing these patients to an increased risk of drug-related problems (DRPs). A prospective, non-randomized, quasi-experimental study was conducted in medical rehabilitation wards to evaluate the impact of pharmacist-delivered interventions and counseling on 30-day unplanned health care utilization and medication adherence for selected rehabilitation patients. A pharmacist provided medication reconciliation and counseling before discharge. Phone follow-up was completed 30 days after discharge to assess for unplanned health care utilization rate and medication adherence. A total of 85 patients (*n* = 43 in prospective intervention group and *n* = 42 in historical usual care group) were included. Among the intervention group, 23 DRPs were identified in 14 (32.6%) patients, resulting in 51 interventions. The intervention group had a significantly lower unplanned health care utilization rate than the usual care group (25.6% vs. 47.6%, *p* = 0.035). The risk of unplanned health care utilization was reduced by over 60% (Odds ratio (OR) = 0.378; 95% CI = 0.15–0.94). Patients reporting medium to high medication adherence increased from 23.6% to 88.4% 30 days after counseling (*p* < 0.05). Pharmacist medication reconciliation and discharge counseling reduced unplanned health care utilization 30 days after discharge and improved medication adherence.

## 1. Introduction

Medication errors and drug-related problems (DRPs) occur frequently upon discharge, as a result of changes in medication regimen or suboptimal discharge instructions given to patients [1,2,3,4,5,6]. Potential DRPs include unintentional medication discrepancies, non-adherence, and adverse drug events, which can cause harm to patients, including side effects, poor disease control, complications, readmissions, and even death [2,3,4,5]. Suboptimal medication management during the transition of care period is a major contributory factor to readmissions [6]. Literature shows clinically important medication errors affected over 50% of patients during the first 30 days after discharge [7,8,9]. A previous local pilot study shows that DRPs occurred in approximately 30% of discharge patients [10]. Among all hospital readmissions, 27% of cases were considered preventable if patients had received appropriate medication monitoring [6].

Patients undergoing rehabilitation generally receive care in several settings before discharge home. Numerous changes in medication regimens likely occur during care transitions, exposing these patients to an increased risk of DRPs. Among all rehabilitation patients, the most vulnerable patient populations are those with complex medical histories, the elderly, and those with polypharmacy which is defined as concurrent use of five or more routine medications [10,11]. Patients with heart failure (HF), acute coronary syndrome (ACS), and stroke are particularly prone to DRPs and readmissions. The 30-day readmission rates across the United States for HF and acute myocardial infarction were on average 24.6% and 19.9%, respectively [12], thereby resulting in a significant clinical and economic burden on the health care system.

Pharmacists play an essential role in identifying and rectifying DRPs while promoting safe and effective use of medications during transitions of care. The positive impact of pharmacist-delivered services, such as patient education, adherence counseling, and medication reconciliation (MR) on improving quality use of medications and reducing preventable DRPs, hospital readmissions, and health care expenditures has been recognized worldwide [13,14,15,16,17,18,19,20]. Therefore, pharmacist-delivered MR and counseling are critical for rehabilitation patients.

Whilst many pharmacist medication review studies have been performed in acute medical or intensive care units, there have been no studies evaluating the impact of pharmacist-conducted MR and discharge counseling for patients undergoing rehabilitation.

The objectives of this study are to investigate the impact of pharmacist-delivered discharge services (MR and discharge counseling) on 30-day post-discharge unplanned health care utilization and to evaluate the impact of discharge counseling on patient medication adherence for rehabilitation patients.

## 2. Materials and Methods

### 2.1. Study Design, Site, and Setting

A prospective, non-randomized, quasi-experimental study was conducted in medical rehabilitation wards in a local public hospital, Tuen Mun Hospital in Hong Kong. The rehabilitation wards involved were for patients transferred from acute care to this unit for longer term physical, occupational, or speech therapy. Patient enrollment began in November 2017 and ended in January 2018. The study was conducted in accordance with the Declaration of Helsinki, and the protocol was approved by the New Territories West Cluster (NTWC) Clinical & Research Ethics Committee (CREC Ref. No.: NTWC/CREC/17094; date of approval: 25 October 2017).

### 2.2. Patient Enrollment

**Figure 1:**
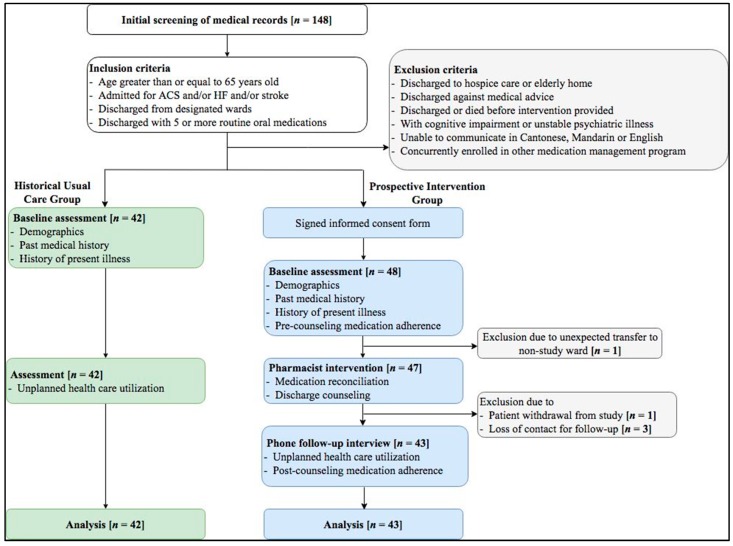
Study schema. ACS = acute coronary syndrome; HF = heart failure.

Eligible patients were recruited if they met all the following inclusion criteria: (1) age greater than or equal to 65 years old; (2) discharged from designated medical rehabilitation wards; (3) hospitalized for ACS and/or HF and/or stroke; and (4) discharged with five or more routine oral medications (defined as chronic medications which need to be taken for at least one year) (Figure 1). 

Patients were excluded if they met any one of the following: (1) discharged to hospice care or elderly home; (2) discharged against medical advice; (3) discharged or died before MR or discharge counseling provided; (4) cognitive impairment or unstable psychiatric illness; (5) unable to communicate in Cantonese, Mandarin, or English; or (6) concurrently enrolled in other medication management program.

### 2.3. Group Allocation

Each eligible subject for the intervention group was approached with or without the presence of a guardian and provided with a study information sheet. For patients agreeing to participate, they gave both written and verbal informed consent to be seen and followed-up by a clinical pharmacist. Patients in the intervention group received baseline assessment, medication reconciliation, discharge counseling, and post-discharge phone follow-up from the pharmacist.

Patients in the historical usual care group were screened using the same inclusion criteria, but they were discharged one month before the enrollment period of the intervention group. They were identified retrospectively using the Clinical Data Analysis & Reporting System for the comparison of 30-day unplanned health care utilization with or without pharmacist discharge services. No direct patient care, neither MR nor counseling, were provided to patients in the usual care group. 

### 2.4. Baseline Assessment

Demographic information and past medical history of all patients were collected from patient or guardian interviews and electronic patient records (ePR). History of present illness, such as principal diagnosis, length of hospital stays, and discharge medications were captured from the ePR, medical chart, and discharge summary. 

For the intervention group, subject pre-counseling medication adherence was assessed using the validated Chinese/English version of 8-item Morisky Medication Adherence Scale (MMAS-8) [21,22]. Total score ranges from 0 to 8, with higher scores indicating better adherence; score < 6: low adherence; score = 6–7: medium adherence; and score = 8: high adherence.

### 2.5. Medication Reconciliation

The intervention group received MR after baseline assessment. A comprehensive patient medication list, including prescribed, over-the-counter drugs, and herbal medications was compiled by the clinical pharmacist by reviewing the drug history profile, clinical notes, and interviewing the patient or caregiver. Discharge orders were checked against the reconciled medication list. If any DRPs were identified, the pharmacist worked with the medical team to resolve the problems and optimize therapeutic outcomes for patients. All DRPs identified were categorized according to the Pharmaceutical Care Network Europe (PCNE) Classification for Drug Related Problems v8.02 [1]. The clinical significance of identified DRPs was assessed by two independent clinical pharmacists with the rating scale proposed by Overhage and Lukes [23]. For those cases with disagreement between the first two pharmacists, a third board-certified clinical pharmacist was consulted to determine the appropriate categorization.

### 2.6. Discharge Counseling

Bedside discharge counseling was provided to the intervention group according to a standard counseling checklist (Appendix A). During the counseling, the pharmacist assessed the patient or caregiver’s understanding of the discharge medication regimen by querying about the uses of each medication and drug schedule to be taken. Information on indication, dosage, and administration of prescribed drugs was given. The pharmacist also counseled on disease management and possible adverse drug effects with managing methods. The differences between pre-admission and discharge regimens were emphasized to enhance medication adherence. Extra counseling was provided for medications with special instructions, such as warfarin, glyceryl trinitrate, or inhalers. Educational leaflets and discharge medication lists were given to patients or caregivers during counseling.

### 2.7. Phone Follow-Up

Phone follow-up was performed for both the intervention and usual care groups. Up to five call attempts were made on different days, primarily at times that each patient had previously indicated as convenient (if any). Any unplanned public or private health care utilization, defined as a composite of first unplanned hospital readmission or emergency department visit within 30 days after discharge, was captured. The effect of pharmacist interventions on unplanned health care utilization was evaluated by comparing the unplanned health care utilization rate between the intervention and usual care group. 

For the intervention group, post-counseling medication adherence was assessed using the MMAS-8 and compared with the pre-counseling value.

### 2.8. Outcome Measures

The primary outcomes were the 30-day unplanned health care utilization rate between the intervention and usual care groups and changes in medication adherence after counseling was provided. The prevalence and clinical significance of DRPs, the acceptance rate of pharmacist interventions, predictors of DRP occurrence, and readmissions were secondary outcomes.

### 2.9. Data Management and Statistical Analysis

Complete-case analysis was applied for this study by excluding participants with missing data. Only patients who received full interventions according to protocol were counted towards the result.

Patient characteristics in both study arms were described using proportions for categorical variables; medians, means, and standard deviations (SD) for continuous variables. Chi-square test and *t*-test were used to test for baseline differences between groups in the distribution of age, gender, ethnicity, prior hospitalizations, principal diagnosis, and medication utilization. Logistic regression was used to examine the impact of pharmacist interventions on the likelihood of 30-day unplanned health care utilization. Patient medication adherence before and after counseling were categorized into groups according to the MMAS-8 and compared using logistic regression. The intervention group was subdivided into “DRP identified” and “Non-DRP identified” groups to identify predictors of DRP occurrence using logistic regression model. Logistic regression was also applied to predict the potential factors associated with readmissions. All data analyses were performed with the Statistical Package for the Social Sciences (SPSS) version 20 (IBM, New York, NY, USA, 2011). Statistical significance was taken as a *p*-value of < 0.05.

## 3. Results

A total of 85 patients were enrolled into the study, of whom 43 patients were in the prospective intervention group and 42 were in the historical usual care group. For the intervention group, five patients with missing data were excluded in the result. The missing data rate was around 10%.

### 3.1. Patient Demographics

Table 1 compares the patient demographics between the intervention and usual care groups. There were no significant differences between the two groups in the baseline characteristics.

### 3.2. Medication Utilization

The total number of drugs was defined as routine medications plus as-needed medications (if any). Table 2 summarizes the medication utilization statistics of study population.

#### 3.2.1. Pre-Admission Medications

Among all recruited patients, 16 (18.8%) patients did not have any routine medications taken before admission. The means of total number of drugs and routine medications taken by the intervention and usual care groups before admission were not statistically significant. The number of patients with polypharmacy was not statistically significant between the two groups.

#### 3.2.2. Discharge Medications

Upon discharge, all patients had polypharmacy due to the requirement of inclusion criteria. The means of total number of drugs and routine medications in the two groups were not statistically significant. 

### 3.3. Identified DRPs and Clinical Significance

A total of 23 DRPs were identified in 14 patients in the intervention group. The prevalence of DRPs was 32.6%, representing 0.53 DRPs identified per patient in the intervention group with no patients having more than three DRPs. The classification of DRPs is presented in Table 3.

#### 3.3.1. (Possible) Causes

According to the PCNE classification, there may be more than one (potential) cause for an identified problem. The most common underlying cause of identified DRPs in this study was related to “drug selection”. The next most common categories were related to “drug use process” and “dose selection”. Furthermore, some DRPs identified were due to “patient related” causes or “no obvious cause”.

#### 3.3.2. Interventions and Acceptance

A total of 51 interventions were performed from the 23 identified DRPs. According to the PCNE classification, interventions are further classified to different levels—prescriber, drug, and patient levels—resulting in the possibility of more than one intervention performed for each DRP. In this study, 2.2 interventions were performed to each identified DRP. 

There is only one level of acceptance from each intervention proposed. Prescribers or patients accepted 48 out of 51 interventions. The overall acceptance rate was 94.1%, and the physician acceptance rate was 91.7%. The acceptance of pharmacist interventions is presented in Table 4.

#### 3.3.3. Medications Implicated in DRPs

The drug classes that were involved in DRPs are classified according to the Anatomical Therapeutic Chemical (ATC) Classification System. Top five classes of medications that are most frequently implicated in the DRPs are gastrointestinal medications (43.4%), cardiovascular medications (17.4%), psychotropic medications (13.0%), analgesics (8.7%), antigout preparations, and musculoskeletal supplements (4.3%). 

#### 3.3.4. Clinical Significance of DRPs Identified

All identified DRPs were rated “somewhat significant” to “significant” with none rated “not significant”. Eleven cases were rated “somewhat significant”, accounting for 48% of all DRPs. The most common underlying causes in this rating were related to the way the patient gets the drugs administered and selection of drugs. On the other hand, 52% of DRPs were rated “significant”. The most common causes were due to selection of drugs (17%) and dosage schedule (13%).

### 3.4. Unplanned Health Care Utilization

Pharmacist intervention was associated with a statistically significant reduction in 30-day all-cause unplanned health care utilization (*p* = 0.035). Among the intervention group, 11 out of 43 patients (25.6%) had all-cause unplanned health care utilization within 30 days after discharge while the rate was 47.6% for the usual care group. The risk of unplanned health care utilization within 30 days was reduced by over 60% in settings with pharmacist interventions Odds ratio (OR) = 0.378; 95% confidence interval (95% CI = 0.15–0.94). Among the readmitted population, the intervention group had a smaller proportion of patients readmitted due to cerebrovascular or cardiovascular diseases when compared to the usual care group (36.4% vs. 40.0%), but the result was not statistically significant (*p* = 0.842; OR = 0.857; 95% CI = 0.19–3.92).

### 3.5. Medication Adherence

**Figure 2:**
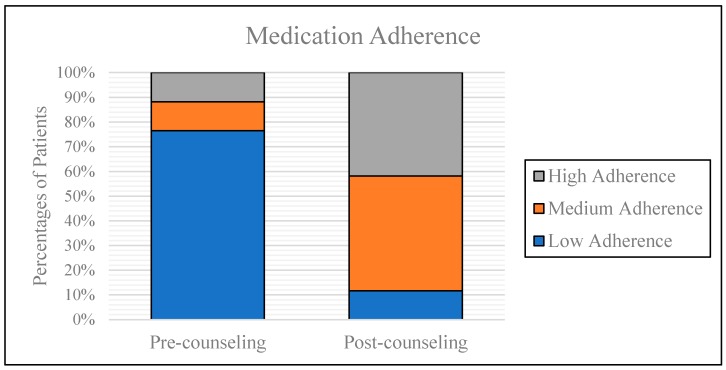
Comparison of pre- and post-counseling medication adherence in the intervention group.

Among the intervention group, nine (20.9%) patients were not taking routine medications prior to admission, so pre-counseling medication adherence assessment was not applicable for them. Majority of patients (76.5%) had low medication adherence before counseling provided while only 11.8% of patients had high medication adherence (Table 5). The means of Morisky score fell under the category of low adherence. After receiving discharge counseling, medication adherence improved in the intervention group. The percentage of patients with high medication adherence significantly increased by over 30% while patients with low adherence significantly decreased by over 60% (Figure 2). 

### 3.6. Risk Factors of Occurrence of DRPs

A logistic regression analysis was performed to ascertain the correlation of total number of pre-admission medications and length of hospitalizations with the occurrence of DRPs. The relationship between the occurrence of DRPs and length of hospital stay was statistically significant. Longer length of stay in hospital was associated with an increased likelihood of experiencing DRPs (OR = 1.042; 95% CI = 1.01–1.08; *p* = 0.016). The relationship between total number of pre-admission medications and the occurrence of DRPs was not statistically significant (OR = 1.107; 95% CI = 0.93–1.32; *p* = 0.247).

### 3.7. Risk Factors Associated with Readmissions

The potential factors associated with readmissions were analyzed by logistic regression. Prior hospitalizations, total number of discharge medications, and pre-counseling medication adherence were put into the model. The relationships between readmissions and all evaluated parameters were not statistically significant (prior hospitalizations: OR = 0.434; 95% CI = 0.02–8.35; *p* = 0.580; total number of discharge medications: OR = 0.952; 95% CI = 0.72–1.27; *p* = 0.734; pre-counseling medication adherence: OR = 0.894; 95% CI = 0.53–1.52; *p* = 0.680).

## 4. Discussion

This study has shown that pharmacist-delivered MR and discharge counseling from a medical rehabilitation ward were associated with a lower rate of unplanned health care utilization and improved medication adherence 30 days after hospital discharge. This highlights the importance of enhancing patient care with pharmacist clinical services and supports the importance of multi-disciplinary health care collaboration. Communication between different professionals allows a more comprehensive evaluation through different aspects and improves holistic patient management.

### 4.1. DRPs and Associated Risk Factors

In this study, 0.53 DRPs were identified per patient in the intervention group. A meta-analysis including 31 studies demonstrates that there were large varieties in the prevalence of DRPs, ranging from 0.13 to 10.6 problems per patient [24]. It is suggested the large range is due to different study designs that some studies only presented the most serious or clinically relevant problems to physicians, whereas some reported all potential DRPs, as was this study design. The training and experience of clinical pharmacists conducting the medication review might also affect the number of DRPs identified. In general, DRPs were quite common during hospitalization, affecting nearly one-third of discharged patients, which is comparable to a previous local study [10]. 

All identified DRPs were rated “somewhat significant” to “significant” independently by the clinical pharmacists. It was found that some DRPs were revealed through patient interviews, for example, patient compliance issues, drug interactions between prescribed and over-the-counter drugs, adverse reactions experienced by patients, and drug storage problems. If there is no clinical pharmacy service to identify these problems, patients may potentially be harmed. This highlights the pivotal role of clinical pharmacists in ensuring medication safety by direct communication with patients or caregivers.

The physician acceptance rate of pharmacist interventions was 91.7% in this study. A meta-analysis reports the physician acceptance rate of pharmacist recommendations varies from 39% to 100% in 31 studies [24]. Many studies described 69% or more as a high acceptance rate [24]. Therefore, this study showed a high acceptance rate of interventions made by the clinical pharmacist. It was likely that the interventions made by the pharmacist were found to be clinically relevant and applicable. In addition, the way in which the clinical pharmacist communicates with the physicians greatly influences the acceptance rate of recommendations. In this study, any DRPs identified, along with possible corrective solutions, were discussed face-to-face with the physicians. If oral discussion was not feasible, putting a written paper note in a conspicuous position of the medical chart was the alternative communication method. This finding is in agreement with previous studies, which show that higher acceptance and implementation rate of pharmacist recommendations was associated with face-to-face contact with the physicians [25] and when more DRPs were revealed through patient interviews [26]. The value of verbal communication in resolving DRPs has been recognized in published studies [27,28]. Clinical pharmacists are encouraged to discuss patient care problems in person with the physicians as well as having close interactions with patients or caregivers.

Length of hospital stay was shown to be a predictor for occurrence of DRPs in this study. Longer length of stay in hospital was associated with higher chance of DRPs, which is consistent with two previously published studies [29,30], but deviates from other findings [31,32]. A previous study revealed that the number of drugs at admission was a risk factor for occurrence of DRPs [33]. However, this study was not consistent with that finding, probably due to the small sample size. By identifying the possible risk factors, patients who are at the highest risk of DRPs should be targeted in settings with limited pharmacy resources.

It is of interest that in a previous local pilot study, prescribing error (necessary information missing) was the most commonly seen DRP [10]. However, no DRPs were identified due to this reason in the current study. With the introduction of In-Patient Medication Order Entry System in the study hospital at the time this study started, computer assistance to enter medication orders in a digital and structured format can greatly reduce prescribing errors of missing necessary information. The most prominent DRPs in this study were related to drug selection, which is consistent with a previously published study [30], but in contrast with another study showing that drug interaction was the most prevalent DRP [29].

### 4.2. Unplanned Health Care Utilization

The all-cause 30-day post-discharge unplanned health care utilization rate was 47.6% in the usual care group, which is much higher than that shown elsewhere (19.6% for US Medicare beneficiaries) [3]. The large difference is likely to indicate higher rehospitalization rates for elderly rehabilitation patients in Hong Kong. This may be explained by two reasons. Firstly, rehabilitation patients usually have chronic illnesses and elderly patients have an even higher risk of readmissions due to polypharmacy and worsening of functional status. Secondly, Hospital Authority is the main statutory body providing low-cost public hospital and emergency services in Hong Kong. When patients feel uncomfortable, they will go to a hospital nearby, leading to a high health care utilization rate.

The all-cause 30-day post-discharge unplanned health care utilization rate was 25.6% in the intervention group, which is comparable to foreign data [3]. Pharmacist clinical service was associated with a statistically significant 60% reduction in all-cause unplanned health care utilization at 30 days. This may be due to resolution of medication discrepancies before hospital discharge, improved drug knowledge, and patient motivation with discharge counseling. This study resulted in a greater treatment effect than that in a previous study (readmissions reduced by 28% at 30 days and 31.9% at 180 days) [34]. This is possibly because of the high baseline readmission rate for this study. 

This study only evaluated the effects over a short period of time, therefore the sustainability of effects could not be judged. A previous randomized clinical trial shows that pharmacist-led interventions reduced 30-day readmissions but did not affect 60-day readmissions [35]. Future studies separating the unplanned health care utilization outcomes into 30, 60, and 180-day post-discharge time frames are warranted.

This study showed that the intervention group had a smaller proportion of patients readmitted due to cerebrovascular or cardiovascular diseases when compared to the usual care group, but the result was not statistically significant. This was probably due to the small sample size in the study.

In addition to evaluating the impact of pharmacist services, this study also explored the potential factors associated with higher readmission rates. However, no risk factors could be identified due to the small sample size. Previous studies have described some risk factors that could potentially increase the risk of readmissions, including prior hospitalizations within 6 months [34]. Future studies focusing on potential factors associated with readmissions are warranted. If the risk factors can be identified, future services may be targeted to patients for whom it would be the most beneficial.

### 4.3. Medication Adherence

The low medication adherence in the majority of patients prior to counseling indicated that there is a strong gap to ask for more resources to prevent readmissions and post-discharge risk to develop iatrogenic adverse outcomes due to poor medication adherence. The patient adherence was notably improved in the intervention group. Clinical pharmacist interventions improved short-term medication adherence. The achieved benefits persisted one month after counseling. This study result indicates that pharmacists are well suited to address the compliance problem and improve patient outcomes [35].

Multiple studies suggest that patients who are not adherent to medication regimens have a poorer prognosis than patients who are adherent [36,37]. Of all drug-related hospitalizations in the United States, 33–69% are the result of medication non-adherence, along with health care costs of approximately $100 billion a year [38]. This study cannot demonstrate a statistically significant association between pre-counseling medication adherence and 30-day readmission risk. However, a published study shows that patients with either a low or an intermediate medication adherence had 2.5 times greater odds of being re-hospitalized within 30 days (*p* = 0.005; 95% CI = 1.3–4.90) [39]. 

Similar to analysis for unplanned health care utilization, as this study only evaluated the effects over a short period of time, the sustainability of pharmacist counseling effect on medication adherence could not be judged. A previous study shows that the persistent benefit could last 12 months after counseling [40]. Future studies with longer follow-up period are warranted.

### 4.4. Limitations

This study did have some limitations. First, this study used a non-randomized design: the usual care group was evaluated retrospectively. Although the baseline characteristics and medication utilization between the two groups found no statistical differences, the existence of clinical differences cannot be ruled out, which may bias our results. There are some confounding factors that could have altered hospital readmissions but cannot be accounted for in our study, for example, different procedures or interventions were performed for different patients during hospitalization. Also, severity of diseases, degree of family support upon discharge, and socioeconomic status could have variations between the intervention and usual care groups, contributing to the readmission rate discrepancy. Ethical considerations were taken into account when a retrospective approach was chosen for the usual care group. It is not ethical for a pharmacist to passively follow the control group prospectively without intervening if they are aware of potentially harmful DRPs.

The second issue was a small sample size that was primarily due to exclusion of patients who were anticipated to discharge to long-term care facilities. The exclusion was judged because there are professional, caring staff in these facilities. If long-term care facility residents are not excluded in this study, it is likely to reduce the differences between control and intervention outcomes, leading to under-estimation of the effect.

Additional limitations include over-reporting of adherence by patients. The MMAS-8 provides pharmacists with a quick assessment of medication adherence since it consists of simple yes or no questions that takes less than a minute to administer. However, like any self-reported measures, the MMAS-8 may have several limitations that may compromise its accuracy, such as recall bias. To avoid bias, future studies may attempt to independently assess the outcomes, for example using pill counts, together with the MMAS-8.

The study design of single site and the absence of sample size calculation are further limitations. The absence of sample size calculation is because of limited resources, only allowing for a small enrollment window.

### 4.5. Implications

This study has several implications in the design and funding of future pharmacy services to improve patient care. Many hospitals may find implementation of clinical pharmacy services difficult because of the expense and limited workforce. It may be possible to design pharmacist-delivered discharge services for patients at particularly high risk. Individual health care systems must determine if the costs saved by the services outweigh the costs associated with the services. A previous study in Sweden demonstrated the cost-effectiveness of clinical pharmacy services with a saving of US$ 1,000,000 per year in health care costs [41]. Pharmacist discharge service is an important strategy that can enhance the safety and quality of care for discharged patients.

## 5. Conclusions

Unplanned readmissions place a huge financial burden on public medical services. Pharmacist MR and discharge counseling reduced unplanned health care utilization in the next 30 days after discharge and improved patient medication adherence. Clinical pharmacists can act as a liaison to bridge the communication gaps between patients, caregivers, prescribers, and pharmacy, thereby providing an overall improvement in quality patient care and health outcomes. Future studies are warranted to focus on optimizing the interventions, identifying patients most likely to benefit from pharmacist involvement, and further explore aspects like patient satisfaction, sustainability of effects of interventions, and patient quality of life.

## Figures and Tables

**Table 1:** Demographic statistics of study population.

Patient Demographics (*n* and %)	Intervention (*n* = 43)			Usual Care (*n* = 42)	*p*-Value ^@^
	DRP Identified (*n* = 14)	Non-DRP Identified (*n* = 29)	Overall (*n* = 43)		
Age: mean ± SD	82.1 ± 8.7	79.7 ± 8.0	80.5 ± 8.2	81.4 ± 8.1	0.597 ^+^
Age: median	82	83	82	81	0.740 ^#^
Female gender	11 (78.6%)	17 (58.6%)	28 (65.1%)	25 (59.5%)	0.595 ^#^
Chinese ethnicity	14 (100%)	29 (100%)	43 (100%)	42 (100%)	/
With prior hospitalizations	13 (92.9%)	26 (89.7%)	39 (90.7%)	33 (78.6%)	0.120 ^#^
Length of hospital stay: mean ± SD (days)	47.8 ± 27.8	27.5 ± 18.6	34.1 ± 23.7	38.2 ± 38.7	0.552 ^+^
Principal diagnosis (*n* and %)					
Stroke	8 (57.1%)	21 (72.4%)	29 (67.4%)	30 (71.4%)	0.690 ^#^
ACS	1 (7.1%)	1 (3.4%)	2 (4.7%)	7 (16.7%)	0.072 ^#^
HF	5 (35.7%)	7 (24.1%)	12 (27.9%)	5 (11.9%)	0.065 ^#^

^@^*p*-Value compares between the overall intervention group and the usual care group. ^+^*p*-value calculated by *t*-test. ^#^*p*-value calculated by Chi-square test. DRP = drug-related problem; ACS = acute coronary syndrome; HF = heart failure.

**Table 2:** Medication utilization statistics of study population.

Medications Used	Intervention (*n* = 43)			Usual Care (*n* = 42)	*p*-Value ^@^
	DRP Identified (*n* = 14)	Non-DRP Identified (*n* = 29)	Overall (*n* = 43)		
Number of pre-admission routine medications					
Mean ± SD	5.1 ± 2.7	4.5 ± 3.8	4.7 ± 3.5	5.1 ± 3.6	0.583 ^+^
Polypharmacy (n and %)	9 (64.3%)	14 (48.3%)	23 (53.5%)	25 (59.5%)	0.575 ^#^
Total number of pre-admission medications: mean ± SD	7.0 ± 3.4	5.6 ± 4.8	6.0 ± 4.4	6.8 ± 5.0	0.456 ^+^
Number of discharge routine medications					
Mean ± SD	7.1 ± 1.7	6.7 ± 2.0	6.8 ± 1.9	7.3 ± 2.9	0.334 ^+^
Polypharmacy (*n* and %)	14 (100%)	29 (100%)	43 (100%)	42 (100%)	/
Total number of discharge medications: mean ± SD	9.9 ± 2.8	8.2 ± 2.8	8.7 ± 2.9	9.3 ± 3.7	0.453 ^+^

^@^*p*-value compares between the overall intervention group and the usual care group. ^+^*p*-value calculated by *t*-test. ^#^*p*-value calculated by Chi-square test.

**Table 3:** Pharmaceutical Care Network Europe (PCNE) classification for DRPs identified in the intervention group.

Primary Domain	Cause	Case (%)	Case (%)
Drug selection: related to the selection of drug	Inappropriate drug according to guidelines/formulary	5 (17.9)	11 (39.3)
	No indication for drug	3 (10.7)	
	No drug treatment despite existing indication	3 (10.7)	
Drug use process: related to the way patient gets the drug administered by a health professional or caregiver, despite proper dosage instructions given	Drug administered via wrong route	9 (32.1)	9 (32.1)
Dose selection: related to the selection of dose or dosage	Dosage regimen too frequent	2 (7.1)	4 (14.3)
	Drug dose too low	1 (3.6)	
	Dose timing instructions wrong, unclear, or missing	1 (3.6)	
Patient related: related to the patient and their behavior	Patient uses/takes more drug than prescribed	1 (3.6)	3 (10.7)
	Patient takes food that interacts	1 (3.6)	
	Patient stores drug inappropriately	1 (3.6)	
Other	No obvious cause	1 (3.6)	1 (3.6)

**Table 4:** Acceptance of pharmacist interventions in the intervention group.

Primary Domain	Implementation	Case (%)	Case (%)
Intervention accepted	Intervention accepted and fully implemented	47 (92.2)	48 (94.1)
	Intervention accepted, partially implemented	1 (2.0)	
Intervention not accepted	Intervention not accepted: unknown reason	2 (3.9)	3 (5.9)
	Intervention not accepted: not feasible	1 (2.0)	

**Table 5:** Comparison of pre- and post-counseling medication adherence in the intervention group.

Adherence Category	Pre-Counseling (*n* = 34)	Post-Counseling (*n* = 43)	Changes	*p*-Value	OR (95% CI)
Low	26 (76.5%)	5 (11.6%)	−64.9%	<0.050 ^#^	0.04 (0.01–0.14)
Medium	4 (11.8%)	20 (46.5%)	+34.7%	0.001 ^#^	6.52 (1.96–21.72)
High	4 (11.8%)	18 (41.9%)	+30.1%	0.004 ^#^	5.40 (1.62–18.04)
Mean ± SD	4.94 ± 1.52	7.08 ± 1.03		<0.001 ^+^	

^#^*p*-value calculated by Chi-square test. ^+^*p*-value calculated by *t*-test. OR = Odds ratio.

## Appendix A

Standard Counseling Checklist

## References

[1] Pharmaceutical Care Network Europe DRP-Classification V8.02. http://www.pcne.org/upload/files/230_PCNE_classification_V8-02.pdf.

[2] Forster A.J., Murff H.J., Peterson J.F., Gandhi T.K., Bates D.W. (2003). The incidence and severity of adverse events affecting patients after discharge from the hospital. Ann. Intern. Med..

[3] Jencks S.F., Williams M.V., Coleman E.A. (2009). Rehospitalizations among patients in the medicare fee-for-service program. N. Engl. J. Med..

[4] Ho P.M., Spertus J.A., Masoudi F.A., Reid K.J., Peterson E.D., Magid D.J., Krumholz H.M., Rumsfeld J.S. (2006). Impact of medication therapy discontinuation on mortality after myocardial infarction. Arch. Intern. Med..

[5] Spertus J.A., Kettelkamp R., Vance C., Decker C., Jones P.G., Rumsfeld J.S., Messenger J.C., Khanal S., Peterson E.D., Bach R.G. (2006). Prevalence, predictors, and outcomes of premature discontinuation of thienopyridine therapy after drug-eluting stent placement. Circulation.

[6] Forster A.J., Murff H.J., Peterson J.F., Gandhi T.K., Bates D.W. (2005). Adverse drug events occurring following hospital discharge. J. Gen. Intern. Med..

[7] Kripalani S., Roumie C.L., Dalal A.K., Cawthon C., Businger A., Eden S.K., Shintani A., Sponsler K.C., Harris L.J., Theobald C. (2012). Effect of a pharmacist intervention on clinically important medication errors after hospital discharge: A randomized trial. Ann. Intern. Med..

[8] Rask K.J., Wells K.J., Teitel G.S., Hawley J.N., Richards C., Gazmararian J.A. (2005). Can an algorithm for appropriate prescribing predict adverse drug events?. Am. J. Manag. Care.

[9] Anderson J.L., Adams C.D., Antman E.M., Bridges C.R., Califf R.M., Casey D.E., Chavey W.E., Fesmire F.M., Hochman J.S., Levin T.N. (2007). ACC/AHA 2007 guidelines for the management of patients with unstable angina/non–st elevation myocardial infarction. J. Am. Coll. Cardiol..

[10] Wong Y.Y. (2017). Pharmacist Clinical Service in an Orthopedic Rehabilitation Ward in A Local Hospital. HKPJ.

[11] Koshman S.L., Charrois T.L., Simpson S.H., McAlister F.A., Tsuyuki R.T. (2008). Pharmacist care of patients with heart failure: A systematic review of randomized trials. Arch. Intern. Med..

[12] Oborne A., Dodds L.J. (1994). Seamless pharmaceutical care: The needs of community pharmacists. Pharm. J..

[13] Modig S., Kristensson J., Ekwall A.K., Hallberg I.R., Midlöv P. (2009). Frail elderly patients in primary care—their medication knowledge and beliefs about prescribed medicines. Eur. J. Clin. Pharmacol..

[14] Krumholz H.M., Merrill A.R., Schone E.M., Schreiner G.C., Chen J., Bradley E.H., Wang Y., Wang Y.F., Lin Z.Q., Straube B.M. (2009). Patterns of hospital performance in acute myocardial infarction and heart failure 30-day mortality and readmission. Circ. Cardiovasc. Qual. Outcomes.

[15] Schnipper J.L., Kirwin J.L., Cotugno M.C., Wahlstrom S.A., Brown B.A., Tarvin E., Kachalia A., Horng M., Roy C.L., McKean S.C. (2006). Role of pharmacist counseling in preventing adverse drug events after hospitalization. Arch. Intern. Med..

[16] Al-Rashed S.A., Wright D.J., Roebuck N., Sunter W., Chrystyn H. (2002). The value of inpatient pharmaceutical counselling to elderly patients prior to discharge. Br. J. Clin. Pharmacol..

[17] Dudas V., Bookwalter T., Kerr K.M., Pantilat S.Z. (2001). The impact of follow-up telephone calls to patients after hospitalization. Am. J. Med..

[18] Szkiladz A., Carey K., Ackerbauer K., Heelon M., Friderici J., Kopcza K. (2013). Impact of pharmacy student and resident-led discharge counseling on heart failure patients. J. Pharm. Pract..

[19] Warden B.A., Freels J.P., Furuno J.P., Mackay J. (2014). Pharmacy-managed program for providing education and discharge instructions for patients with heart failure. Am. J. Health Syst. Pharm..

[20] Kaboli P.J., Hoth A.B., McClimon B.J., Schnipper J.L. (2006). Clinical pharmacists and inpatient medical care: A systematic review. Arch. Intern. Med..

[21] Morisky D.E., Green L.W., Levine D.M. (1986). Concurrent and predictive validity of a self-reported measure of medication adherence. Med. Care.

[22] Wang J., Bian R., Mo Y. (2013). Validation of the chinese version of the eight-item morisky medication adherence scale in patients with type 2 diabetes mellitus. J. Clin. Gerontol. Geriatr..

[23] Overhage J.M., Lukes A. (1999). Practical, reliable, comprehensive method for characterizing pharmacists’ clinical activities. Am. J. Health-Syst. Pharm..

[24] Graabaek T., Kjeldsen L.J. (2013). Medication reviews by clinical pharmacists at hospitals lead to improved patient outcomes: A systematic review. Basic Clin. Pharmacol. Toxicol..

[25] Chen T.F., de Neto A.C. (2007). Exploring elements of interprofessional collaboration between pharmacists and physicians in medication review. Pharm. World Sci..

[26] Jameson J.P., van Noord G.R. (2001). Pharmacotherapy consultation on polypharmacy patients in ambulatory care. Ann. Pharmacother..

[27] Perera P.N., Guy M.C., Sweaney A.M., Boesen K.P. (2011). Evaluation of prescriber responses to pharmacist recommendations communicated by fax in a medication therapy management program (MTMP). J. Manag. Care Pharm..

[28] Doucette W.R., McDonough R.P., Klepser D., McCarthy R. (2005). Comprehensive medication therapy management: Identifying and resolving drug-related issues in a community pharmacy. Clin. Ther..

[29] Maheshkumar V.P., Dhanapal C.K., Ramakrishna R.M. (2016). Outcomes of clinical pharmacist’s interventions in solving drug-related problems in geriatric patients of rural teaching hospital. Pharm. Innov. J..

[30] Wincent M.M., Potrilingam D., Anagha V., Jacob S.C., Andhuvan G. (2017). Assessmet of drug related problems in patients with chronic diseases in the general medicine units of a tertiary care hospital. Int. J. Pharm. Pharm. Sci..

[31] Ayalew M.B., Megersa T.N., Mengistu Y.T. (2015). Drug-related problems in medical wards of tikur anbessa specialized hospital, ethiopia. J. Res. Pharm. Pract..

[32] Tigabu B.M., Daba D., Habte B. (2014). Drug-related problems among medical ward patients in jimma university specialized hospital, southwest ethiopia. J. Res. Pharm. Pract..

[33] Koehler B.E., Richter K.M., Youngblood L., Cohen B.A., Prengler I.D., Cheng D., Masica A.L. (2009). Reduction of 30-day post-discharge hospital readmission or emergency department (ed) visit rates in high-risk elderly medical patients through delivery of a targeted care bundle. J. Hosp. Med..

[34] Ni W.Y., Colayco D., Hashimoto J., Komoto K., Gowda C., Wearda B., McCombs J. (2017). Impact of a pharmacy-based transitional care program on hospital readmissions. Am. J. Manag. Care.

[35] Haynes R.B., Yao X., Degani A., Kripalani S., Garg A., McDonald H.P. (2008). Interventions for enhancing medication adherence. Cochrane Database Syst. Rev..

[36] DiMatteo M.R., Giordani P.J., Lepper H.S., Croghan T.W. (2002). Patient adherence and medical treatment outcomes: A meta-analysis. Med. Care.

[37] Sokol M.C., McGuigan K.A., Verbrugge R.R., Epstein R.S. (2005). Impact of medication adherence on hospitalization risk and healthcare cost. Med. Care.

[38] Osterberg L., Blaschke T. (2005). Adherence to Medication. N. Engl. J. Med..

[39] Rosen O.Z., Fridman R., Rosen B.T., Shane R., Pevnick J.M. (2017). Medication adherence as a predictor of 30-day hospital readmissions. Patient Prefer. Adher..

[40] Taitel M., Jiang J., Rudkin K., Ewing S., Duncan I. (2012). The impact of pharmacist face-to-face counseling to improve medication adherence among patients initiating statin therapy. Patient Prefer. Adher..

[41] Gillespie U. (2012). Effects of Clinical Pharmacists’ Interventions: On Drug-Related Hospitalization and Appropriateness of Prescribing in Elderly Patients. Ph.D. Thesis.

